# Plant Phenolics in the Prevention and Therapy of Acne: A Comprehensive Review

**DOI:** 10.3390/molecules29174234

**Published:** 2024-09-06

**Authors:** Wojciech Koch, Justyna Zagórska, Magdalena Michalak-Tomczyk, Sercan Karav, Anna Wawruszak

**Affiliations:** 1 Department of Food and Nutrition, Medical University of Lublin, 4a Chodźki Str., 20-093 Lublin, Poland; justyna.zagorska@umlub.pl; 2 Department of Animal Physiology and Toxicology, The John Paul II Catholic University of Lublin, Konstantynów 1I Street, 20-708 Lublin, Poland; magdalena.michalak@kul.pl; 3 Department of Molecular Biology and Genetics, Canakkale Onsekiz Mart University, Canakkale 17000, Türkiye; sercankarav@comu.edu.tr; 4 Department of Biochemistry and Molecular Biology, Medical University of Lublin, 20-093 Lublin, Poland; anna.wawruszak@umlub.pl

**Keywords:** acne, phenolics, EGCG, quercetin, nobiletin

## Abstract

Plants are a rich source of secondary metabolites, among which phenolics are the most abundant. To date, over 8000 various polyphenolic compounds have been identified in plant species, among which phenolic acids, flavonoids, coumarins, stilbenes and lignans are the most important ones. Acne is one of the most commonly treated dermatological diseases, among which acne vulgaris and rosacea are the most frequently diagnosed. In the scientific literature, there is a lack of a detailed scientific presentation and discussion on the importance of plant phenolics in the treatment of the most common specific skin diseases, e.g., acne. Therefore, the aim of this review is to gather, present and discuss the current state of knowledge on the activity of various plant phenolics towards the prevention and treatment of acne, including in vitro, in vivo and human studies. It was revealed that because of their significant antibacterial, anti-inflammatory and antioxidant activities, phenolic compounds may be used in the treatment of various types of acne, individually as well as in combination with commonly used drugs like clindamycin and benzoyl peroxide. Among the various phenolics that have been tested, EGCG, quercetin and nobiletin seem to be the most promising ones; however, more studies, especially clinical trials, are needed to fully evaluate their efficacy in treating acne.

## 1. Introduction

Currently, natural products are used increasingly often for the prevention and treatment of many diseases—cardiovascular diseases, metabolic syndromes, cancer, digestive system diseases and neuropsychiatric disorders [1,2,3]. They are also used in the treatment of various dermatological diseases and are an important ingredient in cosmetics [4,5,6]. Plants are a rich source of secondary metabolites, among which phenolics are the most abundant [7,8]. In terms of pharmacological activity and nutritional significance, these are some of the most important compounds present in plants [9]. Phenolics are compounds that have one or more aromatic rings to which one or more hydroxyl groups are directly connected [8]. Of these, the most numerous is a group of compounds called polyphenols. It is a very large family of compounds derived from secondary metabolism in plants whose common feature is the presence of at least two hydroxyl groups attached to the benzene ring. This makes it a very large group that contains both complex compounds with very high molecular weights (over 30,000 Da, such as tannins) and simple substances, such as phenolic acids [7,9,10]. Until now, over 8000 various polyphenolic compounds have been identified in plant species, among which phenolic acids, flavonoids, coumarins, stilbenes and lignans are the most important ones. Polymerized, high-molecular-weight polyphenols, such as tannins and lignins, are also included [9,10]. Of this large group of compounds, over 500 compounds have been identified in food of plant origin, and these are considered dietary polyphenols [11,12]. In addition to their chemical structure, a common feature of these compounds is also their biochemical origin—the shikimate pathway from L-tyrosine or L-phenylalanine [13,14].

## 2. Flavonoids and Phenolic Acids—The Two Most Widespread Groups of Phenolic Compounds

Flavonoids are the most complicated and most widespread group of phenolic compounds. It is estimated that they constitute two-thirds of all polyphenols found in food. The second most common group are phenolic acids, which constitute about one-third of all polyphenolic compounds consumed in food. Therefore, flavonoids and phenolic acids are two classes of polyphenols with the greatest practical importance, both nutritionally and considering natural products used for preventive or therapeutic purposes [7,15].

Flavonoids are the best described and studied group of polyphenols. So far, over 4000 different compounds from this group have been identified, which are responsible for the attractive colors of flowers or leaves and antioxidant activity of fruits and vegetables [10,16]. A common feature of this group is the presence of two aromatic rings (A and B) bound together by a three-carbon-atom oxygenated heterocycle ring (C) (C6-C3-C6). Considering variations in the structure, degree of unsaturation and oxidation of the C ring, flavonoids can be divided into seven subclasses: flavones, flavanones, flavonols, isoflavones, flavanols, chalcones and anthocyanins [9,10,16]. Differences in structure in each group result from the different position and number of hydroxyl groups in the molecule, as well as the degree of alkylation or glycosylation. Quercetin, kaempferol, myricetin, hesperidin, genistein and catechins are some of the most common flavonoids.

Phenolic or phenolcarboxylic acids comprise a group of phenolic compounds having one carboxylic acid group and are found in a variety of plant-derived foods, especially in seeds, the skins of fruits and the leaves of vegetables. They rarely occur as free compounds and most often are bound in the form of amides, esters or glycosides [15,17]. Some strictly termed monophenols, such as *p*-coumaric acid, are included in the group “functional polyphenols” considering that they have properties and characteristics similar to polyphenols [17,18]. They are divided into two subgroups—hydroxybenzoic and hydroxycinnamic acids. The four most abundant simple hydroxycinnamic acids are ferulic, caffeic, *p*-coumaric and sinapic acids, but the most abundant is the combined form of caffeic and quinic acids—chlorogenic acid. Hydroxybenzoic acids derived from benzoic acid can be found in soluble form (conjugated with sugars or organic acids) or in plant cell walls as part of lignins [19]. These compounds are present at low concentrations in red fruits, onions and black radish. The four most widely represented compounds form this group are *p*-hydroxybenzoic, protocatechuic, vanillic and syringic acids [15].

Plant phenolics are well-known to exhibit a variety of functions in plants, including the regulation of growth and development, participation in defense mechanisms and deterring intruders [15]. Their positive role and impact on the human body are also widely known. Many activities of these compounds have been demonstrated and described such as antioxidant, anticancer, antimicrobial, antiallergic, antiviral, hepatoprotective and many more [20,21,22,23]. Many studies have shown the positive effect of phenolic compounds in the prevention of noncommunicable chronic diseases, such as cardiovascular diseases, diabetes, cancer, neurodegenerative diseases and metabolic disorders [7,24,25,26,27,28]. There are also studies indicating antiaging properties of polyphenols, mostly due to their antioxidant activity [9,29]. There are also studies indicating the potential application of various plant phenolics (tea polyphenols, quercetin, apigenin, resveratrol or luteolin) in the treatment of skin diseases, including skin cancer, psoriasis, atopic dermatitis and chronic urticaria [30,31]. Additionally, there is much more research proving the preventive and cosmetic uses of plant phenolics towards different skin and hair imperfections and disorders, such as photoaging, depigmentation, atopic changes and hair loss [4,5,32,33].

Available review articles focus on the positive role of various plant phenolics towards the prevention and treatment of different skin disorders and diseases, in which particular activities are rather generally described. However, there is lack of a detailed review that specifically focuses on the importance of polyphenolic compounds in the treatment of the most common specific skin diseases, e.g., acne. Therefore, the major aim of this review is to gather, present and discuss the current state of knowledge on the activity of various plant phenolics towards the prevention and treatment of acne, including in vitro, in vivo and human studies. The additional aim of this review is to present the results of research on the possibility of combining drugs used in acne therapy with polyphenolic compounds in order to enhance their effect or reduce side effects by reducing the doses. This review primarily presents but is not limited to the newest research, as older interesting and important articles were also included.

This review gathers current knowledge on the potential application of plant phenols in the prevention and treatment of acne vulgaris and rosacea based on in vitro and in vivo (animals and humans) studies. Online databases such as Pubmed, Science Direct, Google Scholar, Web of Science and Scopus were used to search for the scientific data. The keywords that were used to find the relevant data were “acne”, “acne vulgaris”, “rosacea”, “plant phenolics”, “prevention”, “treatment”, “therapy”, “human study”, “animal study”, “in vitro study”, “in vivo study”. The search of the literature was limited to works in English. This review is a collection of studies on the use of plant phenols in the context of the prevention and treatment of acne vulgaris and rosacea published in the years 1981–2024. However, the vast majority of research used in this review was published within the last 10 years.

## 3. Acne—One of the Most Common Skin Conditions

Acne is one of the most commonly treated diseases by dermatologists and other healthcare workers in the United States and worldwide [34,35,36]. Acne vulgaris is a chronic disease that often persists for years [37]. This dermatosis mainly affects young people—35 to even 90% of adolescents suffer from its symptoms [38]. Acne is a disease that involves pilosebaceous units, and therefore skin lesions, which are the main symptom, most often appearing on the face, upper chest and back [39]. Many factors promote the appearance of acne lesions on the skin. The first is abnormal and excessive hyperkeratinization of the ducts of the pilosebaceous units. Excessive keratinization of the infundibular epithelium and the formation of stronger intracellular adhesions by follicular epithelial cells hinders the exfoliation of cells and results in keratinocytes blocking the hair follicle outlets. Another cause of acne development is the excessive production of sebum by the sebaceous glands and seborrhea. Androgens are primarily responsible for stimulating excessive sebum production. Clogged orifices of pilosebaceous units cause sebum to accumulate and remain in the follicles, which causes them to expand. Such conditions are conducive to the multiplication of *Cutibacterium acnes* bacteria in the sebum. This results in an increased number of these bacteria in the pilosebaceous units. Excessive colonization of these microbes promotes perifollicular inflammation. Therefore, increases in the development of *Cutibacterium acnes* and inflammatory processes are additional causes of acne development [40,41,42]. Symptoms of this disease include seborrhea and skin lesions. Skin lesions can be noninflammatory (comedones and whiteheads) or inflammatory—pustules, cysts (also known as nodules) and papules [43,44]. Acne may result in pain, scars, hyperpigmentation or erythema [45].

Various varieties of this disease have been described in the literature: acne vulgaris, acne rosacea, infantile and pediatric acne, acne associated with polycystic ovary syndrome, acne cosmetica, chloracne, drug-induced acne, acne inversa, tropical acne, mallorca acne and acne associated with the use of anabolic steroids (for increasing muscle mass). However, acne vulgaris is the most common type of acne and accounts for 99% of all acne cases [37,46,47,48,49,50,51,52,53,54]. To quantify the severity of acne vulgaris, a five-point scale (ranging from zero to four) is used. On this basis, acne is divided into the follow categories: clear, almost clear, mild, moderate and severe [55]. Severe forms of acne include acne conglobata and acne fulminans (this type occurs mainly in young men) [56].

The surface of the skin is home to many different microorganisms that make up the commensal microbiota. It is formed by a variety of viruses, fungi, bacteria and mites [57,58,59,60]. Its main function is to protect the skin from invasion by pathogenic microorganisms [61]. External factors (lifestyle, hygiene practices, diet and environment) [62,63] and internal factors (a consequence of our genetics) can cause microbiota imbalances [64]. As a consequence, skin conditions such as acne vulgaris and rosacea may develop [43,65,66]. The sebaceous area, which is rich in lipids and has anaerobic conditions, is an ideal habitat for lipophilic bacteria, such as *Cutibacterium* [43,63,64]. One of the main commensal skin bacteria is *Cutibacterium acnes*, which was formerly known as *Propionibacterium acnes* or *Corynebacterium parvum* [67]. It is a Gram-positive anaerobe bacterium. In addition, it is a lipophilic bacterium, so it is mainly found in the sebaceous glands and hair follicles and on the skin of the face and neck. It protects the skin from pathogens by producing antimicrobial substances, metabolizing fatty acids with antibacterial properties and helping to maintain the acidic pH of the skin. However, some subspecies of *Cutibacterium acnes* may contribute to the development of acne under certain conditions [68,69]. In order to maintain consistency with the original results, in this review we used the names of bacteria as in the source publication.

Inflammation is one of the main factors in the development of acne [70]. Interleukin 1 (IL-1), which is a pro-inflammatory cytokine, is suspected to be responsible for the induction of keratinocyte proliferation, as it has been observed to be increased in activity around the uninvolved follicles before the development of hyperkeratinization. In addition, follicular hyperkeratinization may be preceded by the stimulation of the pilosebaceous vasculature resulting from immune-mediated inflammatory processes involving macrophages and CD4^+^ lymphocytes [44]. Compared to healthy skin, increases in mRNA gene levels of the cytokines—interleukin 8 (IL-8), interleukin 10 (IL-10), interleukin 1β (IL-1β) and tumor necrosis factor α (TNF-α)—have been reported in acne-affected skin; mRNA levels are regulated by nuclear factor kappa beta (NF-κβ) and many pro-inflammatory cytokine genes such as IL-8, β-defensin 4, matrix metalloproteinases and granulysin [70]. Enzymes such as lipoxygenase-5 (LOX-5), cyclooxygenase-1 (COX-1) and cyclooxygenase-2 (COX-2) are expressed in sebaceous cells. These enzymes are involved in the metabolism of fatty acids leading to the synthesis of active lipid mediators (15-hydroxyeicosatetraenoic acids, prostaglandins, leukotrienes), which contribute to the development of inflammation. In particular, increased expression of cyclooxygenase-2 was observed in the sebaceous glands affected by acne. In addition, other pro-inflammatory enzymes leading to the development of acne are phosphodiesterases. They induce the preferential expression of pro-inflammatory cytokines (IL-1, -8, -12, -23, TNF-α) because they reduce the intracytoplasmic levels of cyclic adenosine monophosphate (cAMP) [71,72]. Interleukins play a significant role in the pathogenesis of acne. IL-1 induces the remodeling of the pilosebaceous unit and causes the formation of comedones. IL-8 contributes to the influx of neutrophils into inflamed pilosaebaceous units. In turn, IL-12 is produced by monocytes as a response against Gram-positive organisms and causes the expression of defensins (antimicrobial peptides), which are associated with the evolution of acne lesions [73]. Toll-like receptor 2 and toll-like receptor 4 play an important role in the pathogenesis of acne. When these receptors are activated by *Cutibacterium acnes* ligands, pro-inflammatory cytokines are released [74]. In addition, the activation of the toll-like receptor (TLR) contributes to the formation of scars, dermal matrix destruction and acne inflammation because it induces the enzymes responsible for these processes, i.e., metalloproteinases [75].

In addition to inflammatory mediators, hormones play a significant role in the development and course of acne [54]. During puberty, the level of growth hormone increases, and as a result, the amount of sebum secreted also increases, which is due to the growth of the sebaceous glands. The growth of these glands occurs after the stimulation of insulin-like growth factor 1 receptor (IGF-1R), and it is the growth hormone that stimulates the production of insulin-like growth factors (IGF), i.e., a hormone that activates receptors in the sebaceous glands [76]. Other hormones associated with acne are androgens (testosterone, dihydrotestosterone and dehydroepiandosterone sulfate). They regulate genes responsible for sebum secretion and the growth of sebaceous glands; therefore, an increase in the level of these hormones may contribute to the development of the discussed dermatosis [77,78].

The first effects of a specific acne treatment method may appear only after a few weeks. This is because many treatment methods are designed to prevent the appearance of new lesions on the skin rather than cure existing ones [37]. The type of the skin lesions (inflammatory or noninflammatory) and the severity of acne in a given patient are decisive for choosing the appropriate form of treatment [34,79].

Methods of treating acne vulgaris include topical therapies, systemic agents and physical modalities [34]. Common examples of topical therapies are the use of topical antibiotics (TA) (e.g., erythromycin [80], minocycline [81], clindamycin [82] and dapsone [83]), benzoyl peroxide (BP) [84], a combination of BP and TA [83,85,86], topical retinoids (TR) (e.g., tretinoin [87], tazarotene [88], adapalene [89] and trifarotene [90]), a combination of TR and TA/BP [83,91,92,93], salicylic acid [94], azelaic acid [95] and clascoterone [96]. Systemic agents, on the other hand, include systemic antibiotics, isotretinoin [97,98] and hormonal agents [99]. Recommended systemic antibiotics are tetracyclines (e.g., sarecycline [100], minocycline [101] and doxycycline [102]). Representatives of hormonal agents that are recommended for the treatment of acne are combined oral contraceptives [103,104,105] and spironolactone [106,107]. Acne can also be treated using physical modalities (lasers and photodynamic therapy) [34,108], intralesional corticosteroid injections [109] and chemical peels [110,111].

Rosacea is a chronic inflammatory dermatosis that involves the facial skin (nose, cheeks, forehead, chin) and eyes. According to the recent data, 5.5% of the world’s population suffers from this skin disease [112]. The previous literature data indicated a higher prevalence in women than in men, but recent findings show a similar prevalence in both sexes. However, in men, rosacea is usually more severe [46,112,113,114]. It most often affects people with fair skin (white people), but it can also occur in Africans, Asians, African Americans and Latin Americans [114,115]. Ocular symptoms are reported in more than 50% of patients. Ocular symptoms may or may not be accompanied by skin symptoms [116,117,118]. This dermatosis is most often diagnosed in people between 30 and 50 years of age, and it is common in people over 60 years of age. There is also a variant of this disease that occurs in children—pediatric rosacea (it belongs to the rosacea spectrum but has peculiarities compared to the adult subtype) [46,119,120]. Symptoms of rosacea include telangiectasia, persistent or transient erythema, episodes of flushing, (micro)edema and skin lesions (inflammatory pustules and papules, rare phymatous changes), which appear symmetrically in the central part of the face. Rhinophyma, i.e., hypertrophic lesions on the nose, occurs mainly in men [46,121,122].

The standard classification of rosacea was based on symptoms and signs. In this classification, four subtypes of rosacea were distinguished: erythematotelangiectatic, papulopustular, phymatous and ocular [123]. However, in 2017, the National Rosacea Society Expert Committee developed a new standard classification of this dermatosis. This classification system is based on phenotypes (observable characteristics). According to these guidelines, in order to be diagnosed with rosacea, a patient must have at least one of the diagnostic phenotypes (fixed centrofacial erythema in a characteristic pattern that may periodically intensify; phymatous changes) or two of the major phenotypes (flushing; telangiectasia; papules and pustules; ocular manifestations) [114].

The pathogenesis of this disease is based on a combination of neurovascular, immunologic and genetic causes [124]. Many external factors, such as ultraviolet radiation, physical activity, alcohol, temperature changes and spicy food can stimulate transient receptor potential (TRP) channels, especially belonging to vanilloid and ankyrin subfamilies. As a result, the release of vasoactive-neuro peptides, such as calcitonin gene-related peptide, pituitary adenylate cyclase-activating peptide and substance P, is observed, which is responsible for the neurocutaneous pathophysiology mechanisms in rosacea [125].

Moreover, activation of the immune system in the course of this dermatosis can be also caused by microorganisms (various bacteria including *Staphylococcus epidermidis*, *Bacillus oleronius* and Demodex species) [126]. This stimulates proteinase-activated receptor 2 (PAR2) and keratinocyte-derived toll-like receptor 2 (TLR2). Activation of these receptors causes the expression and transformation of cathelicidin (antimicrobal peptide), resulting in angiogenesis and erythema [127]. In addition, toll-like receptor 2 promotes the expression and release of prostaglandins E2, TNF-α, IL-1β and various other angiogenic factors, cytokines, proteases and chemokines. This result in inflammation, telangiectasia and erythema. On the other hand, stimulation of the proteinase-activated receptor 2 causes pain, pruritus and inflammation. These symptoms are also accompanied by a further release of pro-inflammatory chemokines, prostaglandins and cytokines resulting from the recruitment of neutrophils and T lymphocytes and degranulation of mast cells. This, in turn, intensifies inflammation and stimulates further responses from the immune system [122,128,129].

The influence of genetics in the pathogenesis of rosacea is also significant. One study identified single-nucleotide polymorphisms in the butyrophilin-like 2 (BTNL2) and human leukocyte antigen–DRA genes. These genes are associated with the major histocompatibility complex of the acquired immune system. This indicates a key role of immune system regulation disorders in the pathogenesis of rosacea [130].

Topical and oral therapies are used to treat the symptoms of rosacea. Therapy with light devices plays a significant role in alleviating the symptoms of this disease. In addition, the symptoms of this dermatosis can be reduced by proper skin care (the use of gentle cleansing agents, sunscreens and cosmetics that have a positive effect on the integrity of skin barriers) and a proper lifestyle (e.g., avoiding stress, sun exposure, intense exercise, changes in ambient temperature and certain foods) [131,132]. In topical therapy of rosacea skin symptoms, the following are used: retinoids [133], azelaic acid [134], oxymetazoline [131], brimonidine [135], ivermectin [136], clindamycin [121], metronidazole [137] and sodium/sulfur sulfacetamide [131]. On the other hand, agents used in oral therapy are doxycycline (subantimicrobial), azithromycin [121], minocycline [121], doxycycline [138], trimethoprim/sulfamethoxazole [131], isotretinoin [139], clindamycin [131], tetracycline [140], clonidine [133], carvedilol [140] and propranolol [133]. Various devices, surgical interventions and additional chemical agents are also used in the treatment of this dermatosis, including pulsed dye laser, intense pulsed light [114,121], potassium titanyl phosphate [131], erbium, carbon dioxide, cold steel, radiofrequency and electrosurgery [121,133,141,142].

## 4. Antiacne Potential of Plant-Derived Phenolic Compounds—In Vitro Studies

**Figure 1:**
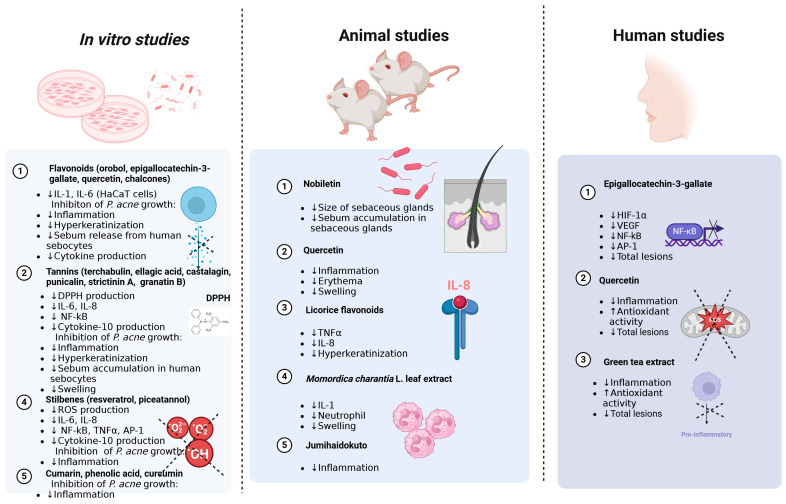
Antimicrobial (inhibition of *P. acne* growth), anti-inflammatory (decrease in expression of inflammation markers), antioxidant (decrease in DPPH and ROS production) and antiacne (decrease in total lesions, size of sebaceous glands, etc.) effects of polyphenols demonstrated in in vitro, animal and human studies. ↑—increase; ↓—decrease. The figure was created with www.biorender.com (accessed on 1 August 2024 by A.W.).

Antibiotics are an effective treatment option for acne caused by microbiological infections. However, their prolonged and widespread use has ultimately led to high levels of antibiotic resistance worldwide [143]. Additionally, an increasing number of scientific studies indicate resistance of pathogenic bacteria to various antibiotics, significantly limiting their effectiveness as a therapy. Prolonged use of antibiotics has led to the emergence of several bacterial resistance mechanisms, including biofilm formation, increasing virulence and spreading resistant strains, as well as genetic mutations, altered efflux pumps and enzymatic inactivation that confer resistance to tetracyclines and macrolides [144,145,146]. In contrast, plant extracts, rich in phenolic compounds, are being successfully tested for their ability to inhibit the growth of bacteria associated with acne, such as *C. acnes*, *S. aureus* and *S. epidermidis* (Figure 1). The antibacterial effects of phenolic compounds are partly linked to their ability to damage bacterial membranes, inhibit virulence factors like enzymes and toxins and prevent the formation of bacterial biofilms [147]. Over the past five years, dozens of articles on natural methods for combating and preventing acne have been published. Currently, several plants and their extracts are already used as natural antiacne agents, including *Aloe vera* L., *Allium cepa* L., *Centella asiatica* L. and tea tree oil [148]. The list of new potential candidates with therapeutic potential against acne continues to grow each year. For instance, Dell’Annunziata et al. [149] tested the effects of bearberry (*Arctostaphylos uva-ursi* L.) leaf extract against *C. acnes*. In the study, the minimum inhibitory concentration (MIC) that caused a bacteriostatic effect on 90% of planktonic *C. acnes* was 0.6 mg/mL. Both MIC and sub-MIC levels affected the biofilm formation stages, with inhibition rates exceeding 50% and 40% at 0.6 mg/mL and 0.3 mg/mL, respectively. *Arctostaphylos uva-ursi* L. leaf extract also disrupted biofilm biomass by 57% and 45% at these same concentrations. Furthermore, the complete inhibition of pro-inflammatory cytokine secretion (IL-1, IL-6, IL-8, TNF-α) was observed when HaCaT cells were cotreated with *Arctostaphylos uva-ursi* L. leaf extract and heat-killed *C. acnes* at concentrations of 1.25 and 0.6 mg/mL. In another recent study by Zöngür [150], extracts from *Beta vulgaris* var. *crassa* were found to be particularly effective at a concentration of 1 mg/mL against bacterial species such as *S. aureus* and *C. acnes*, with the observed effects varying according to the concentration applied. Additionally, Krzemińska et al. [151] tested the antiacne potential of extracts from two *Cotoneaster* species, evaluating their antioxidant, anti-inflammatory and antimicrobial properties, as well as their cytotoxic effects on normal human fibroblasts. Chromatographic analysis using LC-MS revealed that the studied extracts contained 47 compounds, including flavonoids, phenolic acids, coumarins, sphingolipids and carbohydrates. The authors identified several compounds, such as caffeic acid, chlorogenic acid, cinnamic acid and quercetin, which may contribute to antiacne activity. It is believed that phenolic compounds can reduce oxidative stress through several mechanisms largely dependent on their structure. Oxidative stress is considered a significant contributor to the development of acne vulgaris. The overproduction of free radicals plays a role in chronic inflammatory skin conditions like acne vulgaris [152]. For example, flavonoids can scavenge peroxyl radicals, effectively inhibiting lipid peroxidation. They can also chelate redox-active metals, thus preventing the catalytic decomposition of hydrogen peroxide [153].

Considering the richness, variability, complexity and potential interactions of compounds found in plants, a valuable approach is to thoroughly investigate the roles of individual phenolic components to determine their significance in specific biological processes clearly. However, the least numerous studies focus on studying individual phenolic compounds and assessing their roles in reducing infections caused by bacteria involved in the development of acne. This study specifically addresses this group of compounds, including in vitro research.

### 4.1. Flavonoids

The primary and most numerous group of phenolic compounds consists of flavonoids, and the vast majority of research focuses specifically on these compounds. Flavonoids are renowned for their antimicrobial properties, which are closely linked to their chemical structures, particularly the number and arrangement of methoxyl and hydroxyl groups. Research has shown that flavonoids can significantly influence the immune system by supporting the antitumor response, reducing the production of reactive oxygen species and inhibiting inflammatory processes [154]. They are commonly found in plants, primarily in vegetables and fruits. The carbon ring structure of flavonoid molecules facilitates their oxidation in various positions, leading to hydroxylation, methylation and glycosylation. This diversity in substitution and polymerization of functional groups explains the extensive biological functions of flavonoids [32,155].

Isoflavones are natural chemical compounds that belong to the flavonoid group and act similarly to plant hormones. They are commonly found in legumes, such as soy and lentils. The most important isoflavones include genistein, daidzein and glycitein. Isoflavones are often used in cosmetic products, where they help alleviate the effects of skin aging, reduce inflammation and provide proper hydration. Genistein is the most commonly found isoflavone in legumes and has many beneficial health properties, including a positive effect on skin condition. Once absorbed into the body, genistein is converted into various derivatives, such as orobol, which is a rare isoflavone. In a conducted study, it was shown that orobol reduced the production of cytokines IL-1α and IL-6 induced by *P. acnes* in human keratinocytes (HaCaT cell line) in a dose-dependent manner. Moreover, orobol inhibits the growth of *P. acnes*, which may lead to a reduction in inflammation and excessive keratinization caused by these bacteria [156] (Figure 1).

Recently, Alkufeidy et al. [157] examined both green tea extract and the antibacterial activity of catechins alone against the bacteria *S. epidermidis*, *S. aureus* and *C. acnes* isolated from the faces of 30 volunteers. Morphological analysis of the bacterial cells using SEM (scanning electron microscopy) showed that they were distorted and shrunken after treatment with 30 and 40% concentrations of catechins. Additionally, the tested extract caused cell lysis and changes in the morphology of the bacteria, ultimately leading to a reduction in the number of bacterial cells. Epigallocatechin-3-gallate (EGCG), a catechin primarily found in green tea, has shown high efficacy against *C. acnes*, possibly due to complex molecular interactions between EGCG triphenols and bacterial proteins, such as peptidoglycan. These interactions may provide a potential mechanism for its antimicrobial activity. Given that acne is closely related to bacterial overgrowth, inflammation and excessive lipid production, it has been hypothesized that EGCG could be an effective treatment for acne. A study demonstrated that EGCG exhibits apoptotic, sebum-suppressing and anti-inflammatory effects in human sebocytes, suggesting its potential as a therapeutic option for acne [158].

Among flavonols, quercetin appears to be the most prominent representative. Quercetin is a well-known antioxidant and plant polyphenol from the flavonoid group, found in many fruits, leaves and vegetables. The effects of quercetin on inflammatory skin diseases caused by *P. acnes* have been studied both in vitro and in vivo [159]. Kim et al. [160] investigated the antimicrobial potential of *Sanguisorba officinalis* L. roots and first verified the anti-*C. acnes* activity of various fractions of the extracts tested. They then precisely identified the compounds present in this raw material and determined the antimicrobial potential of standards for selected phenolic compounds. The study confirmed that quercetin along with coumarin exhibited the highest antimicrobial activity. Quercetin inhibited the production of pro-inflammatory cytokines in HaCaT cells stimulated by *P. acnes*. In the in vivo study, *P. acnes* was injected intradermally into the ears of mice, causing skin erythema and swelling. Treatment with quercetin significantly reduced ear thickness and swelling. These findings suggest that quercetin may be a potential therapeutic agent against *P. acnes*-induced skin inflammation and could have various pharmaceutical and cosmetic applications [159]. Conversely, kaempferol, ferulic, caffeic, gallic, tannic and benzoic acids, tested in concentrations ranging from 250 to 1000 μg/mL using the disk diffusion method, showed negative results. Coenye et al. [161] investigated extracts from 119 plants used in traditional Chinese medicine for their efficacy against biofilms formed by *C. acnes*. The study revealed that extracts from *Epimedium brevicornum* Maxim. and *Polygonum cuspidatum* Sieb. et Zucc., at concentrations ranging from 0.01 to 0.5% (*w*/*v*), resulted in an almost complete reduction in cell viability within *C. acnes* biofilms. This effect was attributed to the presence of icariin within the extracts. Icariin exhibited a minimum inhibitory concentration (MIC) of 2.5% (*w*/*v*) against biofilms formed by the *C. acnes* strain LMG 16711. Additionally, when icariin was used at sub-MIC levels—concentrations that do not completely inhibit bacterial growth and thereby do not exert strong selective pressure for resistance—it demonstrated statistically significant, concentration-dependent antibiofilm activity.

Chalcones, another group of flavonoids, also exhibit antimicrobial potential against acne. Phloretin, a dihydrochalcone found in strawberries and apples, has demonstrated antimicrobial activity against pathogens such as *Listeria monocytogenes* [162], *Haemophilus influenzae* [163] and *Staphylococcus aureus* [164]. In a study by Kum et al. [165], phloretin at a concentration of 1 mg/mL showed antimicrobial activity against *C. acnes*, *C. granulosum* and *S. epidermidis*, with MIC values of 0.5, 0.5 and 0.25 mg/mL, respectively. Additionally, the experiments indicated that phloretin suppressed inflammatory mediators such as PGE2 and COX-2 in response to *P. acnes*, suggesting that this compound can inhibit the ROS signaling pathway. Cheon et al. demonstrated that phloretin strongly inhibited the growth of *C. acnes* and the Toll-like receptor (TLR) 2-mediated inflammatory signaling in human keratinocytes induced by *C. acnes* [166]. Licorice chalcone A, a chalconoid isolated from the root of *Glycyrrhiza inflate* Bat., demonstrated effectiveness as an inhibitor of NLRP3 inflammasome activation induced by *P. acnes*. This compound blocked the production of caspase-1 (p10) and IL-1β in both primary mouse macrophages and human SZ9 sebocytes. Licorice chalcone A also inhibited ASC speck formation and mitochondrial reactive oxygen species production in response to *P. acnes*. Topical application of licorice chalcone A to mouse ear skin alleviated *P. acnes*-induced skin inflammation, as confirmed by ear thickness measurements and inflammatory gene expression analysis. Additionally, licorice chalcone A reduced caspase-1 activity and IL-1β production in the ears of *P. acnes*-infected mice. The study demonstrates that licorice chalcone A is an effective inhibitor of the NLRP3 inflammasome in controlling *P. acnes*-induced skin inflammation [167].

### 4.2. Tannins

Terchabulin, isolated from *Terminalia laxiflora* Engl., exhibited the highest antimicrobial activity against *C. acnes* among the analyzed compounds, with an MIC value of 125 μg/mL and an MBC value of 250 μg/mL. The study also demonstrated that terchabulin possesses strong antioxidant properties against the DPPH radical and good lipase inhibitory activity. It is believed that the inhibition of lipase activity reduces free fatty acids, leading to the suppression of *C. acnes* growth. Additionally, the study found that ellagic acid showed good anti-*C. acnes* properties in vitro but had inferior lipase inhibitory activity compared to terchabulin [168].

Both *Castanea sativa* Mill. and *C. crenata* Sieb. et Zucc. are sources of compounds that research indicates are effective against acne. For *C. crenata* Sieb. et Zucc., authors identified ellagic acid as the primary compound responsible for its antiacne potential [169]. In contrast, for *C. sativa*, researchers attributed these properties to the ellagitannin castalagin. The research on ellagic acid demonstrated that it inhibited sebum accumulation in sebocytes induced by palmitic acid, a fatty acid known to increase lipid content and trigger inflammatory responses in the skin. Studies on castalagin’s antimicrobial potential revealed that this compound, when tested using the HaCaT cell line, showed promising results [170]. Ellagitannins, known for their antibacterial and anti-inflammatory properties, have been analyzed for their potential in acne treatment. The study focused on chestnut (*Castanea sativa* Mill., *C. sativa*), which is a rich source of ellagitannins, including castalagin. The effects of *C. sativa* Mill. leaf extract and castalagin on human keratinocytes (HaCaT) infected with C. acnes were examined, and it was observed that both compounds inhibited the release of IL-8 and IL-6 at concentrations below 25 μg/mL. The mechanism of action was linked to the inhibition of NF-κB activity, without affecting AP-1 (activator protein 1). Additionally, the extract demonstrated antibiofilm properties and reduced CK-10 (Cytokeratin 10) expression, suggesting its potential role in reducing inflammation, bacterial colonization and keratinization [170].

In a study by Lee et al. [171], punicalagin isolated from *Punica granatum* L. was tested along with three other hydrolyzable tannins—punicalin, strictinin A and granatin B (previously identified in the studied extracts)—against *P. acnes* and *S. aureus* using the disk diffusion method. Among these compounds, punicalagin and punicalin demonstrated the highest inhibitory potential against the tested bacterial strains. For *C. acnes*, the MIC value was 6.25 μg/mL for both compounds, while for *S. aureus*, the MIC value was 12.5 μg/mL. Additionally, morphological changes in bacterial cells were observed after 12 h of incubation with punicalagin and punicalin, including cell shape deformation, swelling and the efflux of intracellular contents.

### 4.3. Stilbenes

Resveratrol, commonly found in grapes and other fruits, is known for its wide range of health-promoting properties. It also possesses scientifically proven antimicrobial potential, including efficacy against acne-associated bacteria. In a study by Taylor et al. [172], microscopic analysis revealed significant alterations in bacterial cell morphology, loss of membrane integrity and disappearance of extracellular fimbrial structures after treating *C. acnes* with resveratrol at concentrations of at least 50 μg/mL. Notably, the bactericidal effect of resveratrol was permanent, unlike the temporary effect of benzoyl peroxide, which was also found to be more toxic to human monocytes and keratinocytes in the same study. The previous literature, including the aforementioned study, reported the antiacne potential of resveratrol [161]. Among over one hundred plant extracts, resveratrol, along with icariin, demonstrated the highest antibiofilm activity. Wei et al. proposed a probable mechanism for resveratrol’s therapeutic effect on acne [173]. Using real-time PCR and enzyme-linked immunosorbent assay analysis with SZ95 sebocytes in vitro, the researchers concluded that resveratrol induces a sebosuppressive and anti-inflammatory effect, partly through the AMP-activated protein kinase (AMPK) pathway. Another plant-derived compound that also belongs to stilbenes, piceatannol, has been shown in studies using *P. acnes*-stimulated keratinocytes to facilitate nuclear factor erythroid 2–related factor 2 (Nrf2) nuclear translocation and target gene transcription, reducing ROS levels. It also inhibited p65 translocation and the secretion of IL-6, TNF-α and IL-8. Additionally, a transfection assay showed that piceatannol reduced *P. acnes*-induced HaCaT cell proliferation and migration by activating the Nrf2 pathway and inhibiting the NF-κB pathway [174]. Stilbenes possess antioxidant, antiproliferative and anti-inflammatory properties. They affect the NF-κB, MAPK and JAK/STAT pathways, reducing the transcription of inflammatory factors and helping maintain homeostatic balance. Resveratrol is the most thoroughly studied stilbene [175]. Resveratrol may be a useful antiacne agent. It is a powerful antioxidant and anti-inflammatory compound that exhibits anticancer and wound-healing properties. It has been shown to inhibit inflammatory markers, AP-1 and NF-κB, which are involved in the formation of inflammatory acne lesions. Resveratrol also has antiviral, antifungal and antibacterial effects and has been shown to inhibit keratinocyte proliferation, contributing to follicular blockage in acne lesion formation, as an alternative to topical treatment for acne vulgaris [172].

### 4.4. Coumarins

Coumarin, extracted from *Sanguisorba officinalis* L. roots, exhibited strong antiacne properties at concentrations ranging from 500 to 1000 μg/mL [160]. Along with quercetin, coumarin was identified as the primary compound responsible for the antimicrobial activity against *C. acnes*.

### 4.5. Phenolic Acids

Few scientific studies have focused on verifying the antiacne effects of phenolic acids in vitro. Gallic acid, found in green tea, wine and other sources, is one such phenolic acid that shows potential as a natural alternative for combating acne. Dos Santos et al. [176] demonstrated that this compound, along with quercetin and resveratrol, exhibits antimicrobial effects against *C. acnes* at a concentration of 5 μg/mL. For comparison, benzoyl peroxide achieved the same effect at a concentration of 1 μg/mL. Mice treated with quercetin, resveratrol or gallic acid demonstrated a 40%, 40% and 30% reduction of the edema, respectively, while mice treated with resveratrol or gallic acid produced a 50 and 45% reduction in IL-1β and a 35% reduction in myeloperoxidase. IL-1, an inflammatory cytokine, is considered to have pathological significance in acne. The canonical NF-κB pathway has been defined in response to IL-1 signaling. *C. acnes* engages the MAPK and NF-κB signaling cascades via the TLR2 receptor on macrophages, thereby modulating the expression of cyclooxygenase-2 (COX-2)/prostaglandin E2 (PGE2) and inducible nitric oxide synthase (iNOS)/NO.

### 4.6. Curcumin

Curcumin is a polyphenolic compound used in medicine due to its anti-inflammatory, antioxidant and antimicrobial properties. Because of these benefits, curcumin is ideally suited for preventing and treating skin inflammations, premature aging, psoriasis and acne. Additionally, it exhibits antiviral, antimutagenic and antifungal effects. Curcumin protects against skin damage caused by prolonged UVB exposure, accelerates wound healing and supports collagen deposition. It also increases the density of fibroblasts and blood vessels in injured areas [177]. The combination of curcumin and the source of the light generated a photodynamic reaction that can target and kill acne-causing *P. acnes* as well as inhibit IL-1 production, providing both antimicrobial and anti-inflammatory effects.

## 5. Potential Application of Plant Phenolics in Acne Vulgaris—Animal Studies

Acne vulgaris is a disease affecting sebaceous glands and pilosebaceous units. In the course of this dermatosis, enlargement of the sebaceous glands and excessive production of sebum are observed [178]. Tretinoin (all-trans retinoic acid (atRA)) has been used in systemic and/or topical treatment of acne vulgaris [179]. One study on hamsters compared the antiacne effects of atRA and nobiletin (a citrus polymethoxy flavonoid). Auricles of 5-week-old male golden hamsters were topically treated once daily with 50 mL of a solution of 1% and 2% nobiletin (1.25 and 2.5 μmol) or 0.2% atRA (0.3 μmol) for 14 days. The control sample consisted of hamsters treated with the vehicle solution alone (95% ethanol and 5% glycerol). It was noted that after treatment with 2% nobiletin and atRA, the size of the sebaceous glands decreased. Since triacylglycerols (TG) are the main components of sebum [180], their amount on the surface of the skin has been studied. It was noted that the relative amounts of TG on the skin surface (% of vehicle treated hamsters) decreased and amounted to 100% (vehicle), 108.2% (1% nobiletin), 66.2% (2% nobiletin) and 35.0% (0.2% atRA). The results obtained in the study provide a basis for considering nobiletin as a potential substance helpful in the treatment of acne [181].

The same study evaluated the influence of nobiletin on the inhibition of sebum accumulation in sebaceous glands and ducts in the UVB-irradiated hamsters. The effect of radiation was investigated since the literature data indicated that UVB causes aberrant functional and morphological changes in pilosebaceous units (hypercornification of keratinocytes, augmentation of sebum synthesis and sebaceous hyperplasia) [182,183]. For this purpose, auricles of 3-week-old male golden hamsters once a day were treated with 2% nobiletin (2.5 μmol) or vehicle after each UVB irradiation (5.4 kJ/m^2^) for 7 days. Sato et al. showed that when exposed to UVB radiation, sebum accumulation was increased in the sebaceous glands and follicular ducts in hamsters. However, when nobiletin was applied topically to the skin of the auricles after each irradiation, it was noted that abnormal sebum accumulation in the sebaceous glands and follicular ducts was inhibited. This is further evidence confirming the antiacne properties of nobiletin [181].

*Propionibacterium acnes* contributes to the development of inflammation in the course of acne because it stimulates the activation of inflammatory cells and secretion of pro-inflammatory cytokines [184]. Therefore, flavonoids with anti-inflammatory properties like quercetin, which can be found in various plants and foods, can be useful in reducing the symptoms of acne [185]. Lim et al. investigated the anti-inflammatory activity of quercetin in mice in which inflammation was triggered by *Propionibacterium acnes*. The mice were divided into five groups (six animals in each group) as follows: control: no treatment; *P. acnes*: live *P. acnes* (1.34 × 10^9^ CFU per 20 μL in PBS); low quercetin: 1 μmol quercetin in 0.01% DMSO per 20 μL of PBS; high quercetin: 10 μmol quercetin per 20 μL of PBS; DEXA: 1 mg/mL dexamethasone per 20 μL of PBS. *P. acnes* (1.34 × 10^9^ CFU per 20 μL in PBS) was intradermally injected into both ears of the mice. After the injection, 20 μL of quercetin in 0.01% DMSO diluted in PBS or dexamethasone was applied to the surface of the skin of the right ear of each mouse [159]. DMSO was a positive control because it has proven anti-inflammatory effects [186]. After the injection of the bacteria, the appearance of swelling, cutaneous erythema and a granulomatous response were observed. Treatment with quercetin (1 μmol and 10 μmol) caused a reduction in swelling, inflammation, erythema, ear thickness and number of inflammatory cells. Based on the obtained results, quercetin can be considered as a natural substance that reduces inflammation caused by *Propionibacterium acnes* and may be useful in the treatment of acne vulgaris [159].

Ruan et al. conducted detailed proteomic studies to explore the mechanisms of licorice flavonoid with antiacne effects in rats. The acne model was established in the back of rats as the model group (MDL), then treated with licorice flavonoid (LCF), while none-treated rats were used as control group (CTR). The study used 6- to 7-week-old male Sprague-Dawley rats. Animals were divided into five groups (eight rats each): experimental (CTR, MDL, LCF), negative control (gel without drug) (NGC) and drug control group (DCG) (without induced acne but treated with the LCF). Acne in rats was caused in the following ways: 0.3 mL of 80% oleic acid once a day was applied to the shaved skin of the back for 14 consecutive days. Next, starting at day 7, 0.1 mL per injection of *Propionibacterium acnes* (1.8 × 10^9^ CFU/mL) was injected into the dermis for 7 days. Then, after modeling, the drug gel patches (with licorice flavonoids—67.3 mg/g) were applied to the back skin for 14 days (once per day, 1 g/100 g, 1 h per time). After the acne was initiated, the skin on the backs of the rats exhibited desquamation, and rough and red-spotted comedones appeared. After applying licorice flavonoids the size of the sebaceous glands decreased, the keratinization was regulated, the infiltration of inflammatory cells was reduced and acne lesions disappeared. The results of the proteomic study showed that transdermal administration of licorice flavonoids could inhibit the PI3K-Akt signaling pathway and mitochondrial activity and thus exhibit antiacne activity. Licorice flavonoids can increase the expression level of FoxO1 (Forkhead box protein O1) in the skin by inhibiting the PI3K-Akt signaling pathway. In addition, the tested substances also promote the phosphorylation of AMPK (AMP-activated protein kinase) to inhibit the biological activity of mTORC1, which finally inhibits the expression of SREBP-1 (sterol regulatory element-binding protein 1). This regulates sebum secretion and inhibits the infiltration of inflammatory cells in the skin. These findings show the antiacne potential of LCF [187].

In another study, the same authors, Ruan et al., examined the antiacne mechanism of licorice flavonoids (LCF) based on the microbiome and metabonomics. The modeling method carried out on Sprague-Dawley (SD) rats was basically in accordance with the report described above [186]. Briefly, the rats were divided into four groups (*n* = 8), namely, the model group (MDL), the control group (CTR), the licorice flavonoids gel group (LCF) and the negative control group (excluding drug gel) group (NGC). After 14 days of modeling (causing acne), the gel was applied to the skin on the mouse’s back for 14 days. Then, rat skin specimens of MDL, CTR, LCF and NGC were taken. In the study, it was observed that LCF can reduce hyperkeratosis and inhibit the inflammatory response in acne (reducing the expression level of IL-8 and TNF-α in serum and in the skin). In addition, analyses have shown that licorice flavonoids can be used in acne therapy because they are helpful in maintaining the metabolic balance of lipids, fatty acids and amino acids in the skin and serum. In addition, the studied flavonoids can restore the normal composition of the skin microbiome, which is disturbed in acne-affected skin. Based on the study, it can be concluded that licorice flavonoids exhibit antiacne effects by regulating microbiological and metabolic balance [188].

Other researchers have also induced acne in an animal model by intradermal injection of *Propionibacterium acnes*. Huang et al. investigated the effect of total phenolic extract of *Momordica charantia* L. leaf (TPE) on inflammation caused by these bacteria. The experiment was performed on 8-old male ICR mice, which were divided into groups of five individuals. *Propionibacterium acnes* was intradermally injected into the left ear of each mouse (6 × 10^7^ CFU per 10 μL in phosphate-buffered saline, PBS) and the right ear was injected with 10 μL of PBS. Next, 10 μL of luteolin or TPE in 5% DMSO in PBS was injected into the same location of both ears right after PBS or *P. acnes* injection. After 24 h, the increase in ear thickness was measured. The intensity of edema was evaluated by the weight difference between the left and the right ear disks. The increase in ear weight and thickness (showing inflammation) after bacterial injection was calculated and expressed as a percentage of the PBS-injected control. The results showed that TPE significantly attenuated *P. acnes*-induced ear swelling in mice along with microabscess. Both luteolin and TPE significantly reduced *P*. *acnes*-induced ear swelling (determined by ear thickness and ear biopsy weight) [189].

Within the same study, a separate experiment was conducted. Mice (*n* = 5) were intradermally injected in the ear with PBS, *P. acnes*, *P. acnes* + luteolin or *P. acnes* + TPE, as described above. Twelve hours after the initial injection, the ears were excised. Then, the samples were analyzed using flow cytometric analysis. Injection of the bacteria caused inflammation, increased IL-1β^+^ concentration and increased total CD45^+^ leukocyte and neutrophil (CD45^+^Ly6G^+^) infiltration. Flow cytometry analysis revealed that application of the total phenolic extract of *Momordica charantia* L. leaf significantly decreased the level of interleukin IL-1β^+^ and total CD45^+^ leukocyte and the migration of neutrophils. Furthermore, it was noted that TPE treatment attenuated the microabscess response to *Propionibacterium acnes*. Obtained results indicated that the epicutaneous application of *Momordica charantia* L. extract rich in phenolics (gallic, chlorogenic, ferulic, caffeic and cinnamic acids, myricetin, quercetin, luteolin, apigenin and thymol) effectively suppressed *P. acnes*-induced inflammation [189].

Matsumoto et al. investigated the effect of the oral administration of Jumihaidokuto (JHT) on *P. acnes*-induced inflammation in the skin of rats. The study also included a quantitative and qualitative analysis of polyphenols in the JHT extract (LC-MS/MS analysis showed the presence of numerous polyphenols). Jumihaidokuto is a pharmaceutical-grade traditional Japanese (kampo) medicine and has been used widely for the treatment of skin diseases including inflammatory acne [190]. The 7- to 8-week-old male Sprague-Dawley rats were intradermally injected with *Propionibacterium acnes* (0.14 mg/50 μL) or saline (control) into the ventral side of both ears. JHT was orally administered to rats at 0.5 g/10 mL/kg in distilled water 1 h before and 6 h after the bacterial injection. Prednisolone (PDN) was used as a reference drug and was orally administered to rats at a dose of 10 mg/10 mL/kg in distilled water. The ear thickness was measured at 0 and 24 h after the injection. All data on the increase in ear thickness were expressed as a percentage of the previous value in each individual rat. Rats subjected to intradermal injection of *P. acnes* and orally administered distilled water developed rapid cutaneous erythema and ear swelling, which were indicative of inflammation. In addition, there was a 181% increase in ear thickness in a 24 h period compared with that prior to injection. In turn, in animals treated with 0.5 g/kg JHT, only a 132% increase was observed. Prednisolone (PDN) exhibited a 123% increase. Based on additional analyses, the authors concluded that Jumihaidokuto inhibits skin inflammation through the antioxidant effects of its various metabolites (including liquiritigenin 7-O-glucuronide and genistein 7-O-glucuronide) [191].

A summary of the potential use of plant phenols in acne vulgaris using in vivo animal studies is presented in Table 1.

## 6. Potential Application of Plant Phenolics in Various Types of Acne—Human Studies

Most of the studies conducted on the possibility of using phenolic compounds in various types of acne are in vitro studies or studies on laboratory animals. There are few studies conducted on humans. In general, green tea extracts, rich in polyphenolic compounds called catechins, are widely used in skin care and dermatological dysfunctions, mostly in the form of different cosmetics [5,194]. Domingo et al. conducted a small randomized, double-blind, split-face trial using a cream containing 2.5% *w*/*w* of EGCG (epigallocatechin-3-gallate)—a major constituent of green tea. The study was conducted on four healthy volunteers with typical rosacea symptoms like significant erythema and telangiectasia. The cream was applied on part of the face, twice daily for six weeks, while the vehicle control was applied on the other part of the face. Immunohistochemistry results revealed a significant decrease in VEGF (vascular endothelial growth factor) and HIF-1α (hypoxia inducible factor-1-alpha) expression, which resulted in a significant reduction of telangiectasias on the face part treated with EGCG in comparison to control (13.8% vs. 28.4% of the epidermis with erythema). VEGF, a key factor in angiogenesis, binds to VEGF receptor 2 (VEGFR2) on endothelial cells, promoting their proliferation, migration and survival. HIF-1, consisting of HIF-1α and HIF-1β subunits, is regulated by oxygen levels. Under hypoxic conditions, HIF-1α accumulates and forms a complex with HIF-1β, which binds to the hypoxia response element (HRE) in target genes, promoting VEGF expression. VEGF and HIF-1α are both crucial in the regulation of angiogenesis, erythropoiesis and iron metabolism, and therefore, the reduction of their expression may result in reduced redness and telangiectasia observed in rosacea. The study, although conducted on a very small number of patients, showed the possibility of using cosmetics containing green tea polyphenols in rosacea, especially in the prevention of telangiectasias [194,195].

Vascular changes and increased vascular wall permeability is a major factor in the pathogenesis of rosacea. Therefore, substances that reduce capillary permeability are effective in treating the symptoms of rosacea. Rigopoulos et al. performed a randomized placebo-controlled trial of a flavonoid-rich *Chrysanthellum indicum DC.* extract. This plant-derived natural product, characterized by high concentrations of phenylpropenoic acids, flavonoids and saponosids, was administrated topically to 125 patients with moderate rosacea twice daily for twelve weeks, in comparison to the placebo group (121 patients). The treatment applied resulted in the reduction of erythema and overall severity of rosacea in comparison to the control group. Moreover, adverse reactions were mild and did not differ to the control. Although the investigated group was large, the exact composition of the investigated extract was not determined, so the potential influence of particular compounds was not evaluated [196].

The majority of studies regarding the potential activity of various phenolics towards Acne vulgaris are mainly in vitro tests of antibacterial activity towards the bacteria responsible for the development of this disease, as well as the potential to extinguish the inflammatory response, which was already described above. There are only a few human studies, which proved the potential of the application of phenolic substances in the treatment of Acne vulgaris. Increased sebum production has a significantly negative effect on acne lesions; therefore, isotretinoin and hormonal therapy are the most commonly used to reduce its production. Yoon et al. studied the potential application of EGCG for the same reason, both in vitro using human SEB-1 sebocytes and in vivo in an 8-week randomized, split-face, clinical trial. The randomly chosen affected areas on the half of the face of the 35 patients (17 men and 18 women) were topically treated with 1 or 5% of EGCG solution, whereas the affected areas of the opposite side were treated with vehicle control (only 3% of ethanol). Positive results from the in vitro study were proved in a clinical trial. EGCG reduced mRNA and protein levels of IL-1α, IL-1β, IL-8, TNF-a, MMP-2, MMP-9, phospho-c-Jun and phospho-IκB in SEB-1 sebocytes and inhibited NF-κB p65 nuclear translocation. It also decreased IL-1α, NF-κB and phospho-IκB in HaCaT keratinocytes. The effects were reversed by compound C (a selective chemical inhibitor of AMPK), indicating the involvement of the AMPK pathway. EGCG thus reduces the inflammation in SEB-1 and HaCaT cells through the AP-1 and NF-kB signaling pathways. It was revealed that topical treatment with EGCG improved both inflammatory and noninflammatory acne lesions, with few, mild side effects. It was proved that topical therapy with EGCG may be rapid and effective therapy of Acne vulgaris. It was also suggested that EGCG may be especially effective on acne lesions connected to hair follicles [158]. This suggests that natural products like plant phenolics may be effective in the treatment of acne in comparison to classical drugs, including antibiotics, benzoyl peroxide, retinoids or hormones. Polyphenolic compounds may be especially useful in treating acne caused by bacterial strains resistance to common antibiotics, like clindamycin, erythromycin or lymecycline. They can also reduce the occurrence of serious side effects, such as those seen with oral retinoids or hormones. However, it should be remembered that their effectiveness may be limited, especially in the case of severe acne lesions or those caused by multidrug-resistant bacteria. The latest recommendations for acne treatment pay special attention to the growing resistance of bacteria to currently used antibiotics. Therefore, it is recommended to move away from monotherapy in favor of combined therapies. Attempts to combine polyphenols with currently used drugs may prove valuable and promising here, which will be discussed later [197].

Not only the type of chemical compound but also the pharmaceutical form in which it is administered may be of great importance in effective acne therapy. Amer et al. evaluated the effectiveness of a novel nutraceutical—quercetin in vitamin C-based nanovesicles called aspasomes—in the treatment of Acne vulgaris among 20 patients (14 females and 6 males). A thin layer of the quercetin aspasomal formulation (100 μL corresponding to a quercetin dosage of 100 μg) was applied once daily directly on affected areas on the right side of the face. On the opposite side of the face, panthenol formulation was applied (placebo group). The study was conducted for 12 weeks. This exploratory clinical trial confirmed the effectiveness of quercetin-loaded aspasomes, which resulted in a significant reduction in inflammatory, noninflammatory and total acne lesions. The observed decreases in inflammatory changes were 77.9, 11.8 and 55.3%, for inflammatory lesions, comedones and total lesions, respectively. An additional in vitro assay confirmed the increased antioxidant, anti-inflammatory and antibacterial efficacy on *Propionibacterium acnes* of this novel nutraceutical in comparison to quercetin alone. High skin deposition (reaching 40%) because of the small molecule diameter (125–184 nm) and negative charge with good storage stability were also confirmed [198]. This shows that modern pharmaceutical drug delivery systems such as liposomes, hydrogels or microneedles can effectively increase the effectiveness of the drugs used, including natural products. This applies to both general and locally applied substances [199].

Lu and Hsu evaluated the potential of oral EGCG supplementation in improving acne in a group of postadolescent women in a randomized, double-blind and placebo-controlled clinical trial. The study was conducted in a group of 80 subjects with acne vulgaris receiving 1500 mg of decaffeinated green tea extract (57.12% *w*/*w* of EGCG) or placebo (cellulose) daily for 4 weeks. The investigated extract was administered in the form of a capsule 30 min after meals 3 times per day. The study revealed significant improvement in inflammatory lesion counts distributed on the nose, periorally and on the chin of patients with moderate to severe acne in comparison to the placebo group. However, there were no significant differences in the number of total lesions. In the group that received green tea extract, a significant decrease in inflammation and total lesions count on the forehead and cheek was observed. Additionally, a significant decrease in the total cholesterol level of patients receiving green tea was also observed [200]. Available clinical trials using plant phenolics are summarized in Table 2.

## 7. Synergistic Effect of Polyphenols with Synthetic Drugs in the Treatment of Acne

Acne vulgaris is a common chronic inflammatory disease with a complex pathogenesis. Although there are many ways to treat acne, current therapies may be inadequate due to limited efficacy, low tolerance or high costs for patients [201]. Typical acne treatment methods include topical medications, oral antibiotics, retinoids and hormonal therapies. However, medicinal plants are increasingly being used as well [194]. Natural plant compounds, known as phytochemicals, are gaining increasing interest as alternative treatment methods due to their low toxicity. Their use in dermatology is, however, limited by their easy degradation when exposed to high temperature, light and oxygen, as well as their poor penetration through the skin barrier. A common method for delivering drugs and phytochemicals is encapsulation in lipid nanoparticles, which allows effective delivery of appropriate concentrations of these substances to the target site with minimal side effects [202].

Various phenolics can be combined with synthetic or semisynthetic drugs to achieve a synergistic effect and increase their activity against acne or decrease the drug dosage. The majority of the available data are from in vitro studies using different chemotherapeutic drugs, which are commonly used in the treatment of Acne vulgaris. An in vitro study was performed to evaluate the effectiveness of combining two flavonoids, quercetin and kaempferol, with two commonly used antibiotics, eythromycin and clindamycin, against antibiotic-resistant *P. acnes*. The study was conducted in two models of both flavonoids separately and in combination with antibiotics. The study revealed a significant increase in bactericidal activity when kaempferol or quercetin were combined with clindamycin rather than with erythromycin. Both phenolics were active against bacterial strains. The minimum inhibitory concentrations (MICs) were ≤32 μg/mL and ≤64 μg/mL for quercetin and kaempferol, respectively. However, the combination of both phenolics did not show any synergistic effect in contrast to that observed when these two natural compounds were combined with clindamycin [203].

Very recently, Alkufeidy et al. analyzed the antimicrobial properties of catechins at two concentrations (0.3 g/mL and 0.4 g/mL) in combination with antibiotics (tetracycline, erythromycin and clindamycin) against three acne-associated bacterial species: *S. aureus*, *S. epidermidis* and *C. acne.* These bacteria were isolated from the facial surfaces of acne sufferers for the experiment. The study used the well diffusion method and found that the antibacterial activity of catechins was concentration-dependent. Moreover, when combined with antibiotics, the catechins exhibited maximum synergistic activity [157].

A study conducted by Taylor et al. evaluated the efficacy of combining benzoyl peroxide and resveratrol on the collective strain *C. acnes* [172]. It was noted that resveratrol achieves a critical concentration around 50–75 μg/mL, where it surpasses a threshold leading to significant growth inhibition and sustained bactericidal effects. Conversely, benzoyl peroxide exhibited strong bactericidal activity initially, but this effect did not persist beyond the first 24 h. When resveratrol and benzoyl peroxide were combined, the strong bactericidal effect of benzoyl peroxide, along with the high inhibitory activity of resveratrol, led to consistently low levels of bacteria throughout the experiment. The authors emphasized that the combination of these compounds can be complementary in vivo, resulting in improved clinical outcomes. Additionally, a previous study found that combining benzoyl peroxide with antibiotics helps reduce the bacteria’s ability to develop drug resistance [204].

The effectiveness of combining the stilbene aglycone metabolite of rhaponticin, rhapontigenin, with clindamycin was tested against antibiotic-resistant *C. acnes* [205]. The study, based on checkerboard assay results, found that the combination of the clindamycin and tested stilbene inhibited *C. acnes* growth at a concentration of 4 μg/mL, whereas clindamycin alone required a concentration eight times higher to achieve the same effect.

Extensive research has been conducted on gold nanoparticles capped with various antibiotics and compounds to assess their antibacterial activity. Lambrechts et al. investigated the effectiveness of gold nanoparticles combined with rosmarinic acid in combating biofilm formation by *C. acnes* and *S. epidermidis*, both of which contribute to wound formation. Gold nanoparticles synthesized with rosmarinic acid significantly enhanced wound closure by 21.4% at a concentration of 25% compared to both the negative cell control and rosmarinic acid alone at its highest tested concentration of 500 μg/mL. Nevertheless, no antibacterial activity was observed for this combination at the mentioned concentration. This reduction in activity may be attributed to decreased stability of these nanoparticles in brain heart infusion (BHI) broth, as indicated by the stability studies [206].

In the case of acne lesions, excessive sebum production has a particularly negative impact on the patient. To reduce its secretion, isotretinoin and hormonal therapy are usually used. An alternative is EGCG, which is the main phenolic component of green tea. Studies have shown that EGCG reduces sebum production by modulating the AMPK–SREBP1 signaling pathway. Additionally, EGCG has anti-inflammatory effects as it inhibits the NF-κB and AP-1 pathways, induces apoptosis of sebocytes, leading to their cytotoxicity, and reduces the viability of *P. acnes*, thereby positively affecting most pathogenic aspects of acne. In clinical studies, EGCG improved the appearance of acne lesions and was well tolerated by patients [158].

The antiandrogenic properties of plant extracts are also utilized in acne treatment. An aqueous extract of Japanese cherry bark has shown the ability to bind to the estrogen receptor beta. In this extract, sakuranetin, naringenin, genistein, genkwanin and arctigenin were analyzed, with genistein showing the strongest binding ability [194].

## 8. Conclusions and Future Perspectives

Acne is one of the most frequently diagnosed skin diseases. It is a chronic condition, the proper treatment of which lasts for years and requires the use of many different agents applied both locally and generally. The disease is mainly inflammatory, very often complicated by infection with *Cutibacterium acnes*. Many different types of acne are classified, of which acne vulgaris and rosacea are the two most frequently diagnosed types. Phenolic compounds, due to their considerable diversity and high biological activity, have found a special place in studies of their potential use in acne therapy. A particularly large number of studies have been conducted in the last 5 years which have shown the high effectiveness of many phenolic compounds in the eradication of bacteria responsible for the development and progression of acne lesions, also taking into account antibiotic-resistant strains. These are mostly in vitro studies, and among the tested phenolic compounds, the most positive results were obtained for simple polyphenols—EGCG and quercetin, as well as condensed tannins such as terchabulin or castalagin. Promising results were also obtained for two stilbenes—resveratrol and piceatannol. Because of its strong antioxidant, antibacterial, antiviral, sebosuppressive and anti-inflammatory effect, resveratrol seems to be one of the most promising antiacne natural agents. The focus on phenolic compounds like EGCG, quercetin and nobiletin stems from their recognition as some of the most promising agents in acne therapy. These compounds were selected due to their proven efficacy in multiple studies, showing strong anti-inflammatory, antioxidant and antibacterial effects, which are critical in managing acne. EGCG, for example, has been shown to regulate sebum production, while quercetin and nobiletin are noted for their significant anti-inflammatory properties. Additionally, these compounds are frequently referenced in the literature, which highlights their widespread acceptance and reliability in research.

However, it is important to note that there are many other phenolic compounds that also demonstrate effectiveness in combating acne. Studies have shown that a variety of phenolics can offer similar benefits, but they may not have been as extensively studied or featured as prominently in research to date.

It should be remembered, however, that the vast majority of research comprises in vitro studies, most often conducted on cell lines or assessing the effectiveness of natural substances in relation to pathogens involved in the development of acne. These studies have revealed that plant polyphenols may reduce inflammation by decreasing IL-1 and IL-6 and IL-8 production, hyperkeratinization and sebum release. Many in vitro research studies have also found that plant phenolics significantly reduce ROS production, which is an important factor in limiting the development of acne lesions. However, it seems that the most important conclusion from in vitro studies is related to the direct antibacterial action of phenolic compounds against the bacteria responsible for the development of acne lesions, especially *P. acne*. There are definitely fewer studies conducted on animals, and they only concern the assessment of effectiveness in acne vulgaris. Nobiletin and quercetin, as well as licorice flavonoids, have shown high effectiveness by reducing the size of sebaceous glands, sebum accumulation, inflammation, erythema and swelling. There are also studies where positive results of in vitro experiments on the reduction of acne lesions were subsequently confirmed using animal models. However, there are no results of studies on phenolic compounds in rosacea conducted in an animal model in the literature.

It is important that positive results were obtained for some phenolic compounds in clinical studies both in acne vulgaris and in rosacea. Here, significant effectiveness was demonstrated for green tea extracts, as well as for individual substances such as EGCG (reduction of HIF-1α, VEGF, NF-κB and AP-1 activity and total lesions) or quercetin (reduction of inflammation, ROS production and total lesions). It is interesting that the results of studies from in vitro experiments, on animals and on humans are similar. They are based on similar mechanisms and lead to similar effects in reducing acne lesions. It seems, therefore, that in this case, the use of the in vitro model may prove to be cheaper, faster and more ethical compared to conducting studies on animals. A graphical presentation of the results of studies conducted in the in vitro model, in vivo and on humans, taking into account the substances and mechanisms used, is presented in Figure 1.

It seems unlikely that therapy for this disease in humans would be initiated using only phenolic compounds. However, studies performed on animals revealed that some phenolic compounds can be as effective in treating acne as standard medications—topical or systemic tretinoin or topical steroids such as dexamethasone or prednisolone, which were mainly used to evaluate the comparative anti-inflammatory effects of polyphenolic compounds. Especially promising results were obtained in the case of antibiotic-resistant strains of *P. acnes*, where quercetin or kaempferol were equally effective in comparison to clindamycin or erythromycin [203]. Plant phenolics may also have significantly different antibacterial kinetics in comparison to standard antiacne drugs like benzoyl peroxide. Taylor et al. [172] revealed that although bactericidal activity of resveratrol against *P. acnes* was lower in comparison to BP, its action was much longer. The strongest antibacterial activity of benzoyl peroxide was observed only during the initial 24 h, and then it started to decline rapidly. In contrast, the activity of resveratrol against *P. acnes* slowly increased for up to 10 days. Therefore, the most beneficial solution was to combine both substances, which provided stronger and longer antibacterial activity compared to monotherapy. It should be remembered, however, that the authors of the aforementioned studies strongly emphasize that their promising results were achieved only in the in vitro model and that many additional studies are needed to introduce such therapeutic strategies into clinical practice. It seems more likely that common drugs with known antiacne activity could be combined with natural polyphenols to increase the effectiveness of the therapy, especially in inflammation involving drug-resistant bacteria. Promising results were obtained in this case for EGCG, quercetin, kaempferol or resveratrol, which were used together with commonly used drugs such as clindamycin, erythromycin or benzoyl peroxide. However, these types of combined therapies require more research to develop optimal doses, identify potential side effects and determine the sensitivity of antibiotic-resistant bacterial strains. It also seems reasonable to search for new plant phenolic compounds with antiacne potential. Since conducted experiments were only in vitro, studies using animal models, and especially clinical studies, are needed to fully confirm the preliminary obtained effects of combining drugs with polyphenolic compounds and to be able to introduce such therapy into clinical practice. A literature search revealed that there are only a few clinical trials evaluating the efficacy of plant phenolics in the treatment of acne and that there are no new ongoing studies of this type. The clinical trials mentioned, although they showed promising results, were conducted on a limited number of patients, and only one study enrolled more than 100 people. This shows that there is a need not only for further studies in this area but also for fully randomized studies on a large number of patients using the rigorous criteria currently required in clinical trials to fully assess the potential (dose, safety profile, efficacy) of the most promising phenolic compounds in the treatment of acne.

## Figures and Tables

**Table 1:** Application of plant phenolics in acne vulgaris—animal studies.

Compound/Extract	Natural Sources	Study Model	Dosage	Results/Effect	References
			**Acne vulgaris**		
Nobiletin	Citrus fruits (mandarin oranges, tangerines, oranges, grapefruits)	Auricles of 5-week-old male golden hamsters	Topical application (solution) 1% and 2% (1.25 and 2.5 µmol)	Size of sebaceous glands ↓ TG ↓	[181,192]
Nobiletin	Citrus fruits (mandarin oranges, tangerines, oranges, grapefruits)	Auricles of 3-week-old male golden hamsters	Topical application (solution) 2% (2.5 µmol)	Sebum accumulation in sebaceous glands and ducts (after the UVB-irradiated) ↓	[181,192]
Quercetin	Tea, onions, apples, lettuce, broccoli	Ears of 6-week-old male BALB/c mice (*n* = 8/group)	Topical application (solution) 1 and 10 µmol	Ear thickness ↓ Inflammation ↓ Erythema ↓ Swelling ↓ Inflammatory cells ↓	[159,193]
Licorice flavonoids	Licorice (*Glycyrrhiza glabra* L.)	Back skin of 6- to 7-week-old male Sprague-Dawley rats (*n* = 8/group)	Topical application (drug gel patches) 67.3 mg/g	Size of sebaceous glands ↓ Hyperkeratinization ↓ Infiltration of inflammatory cells ↓ Acne lesions ↓	[187]
Licorice flavonoids	Licorice (*Glycyrrhiza glabra* L.)	Back skin of 6- to 7-week-old male Sprague-Dawley rats (*n* = 8/group)	Topical application (drug gel patches) 67.3 mg/g	Hyperkeratinization ↓ TNF-α ↓ IL-8 ↓ Metabolic balance Microbial balance	[188]
Total phenolic extract of *Momordica charantia* L. leaf	*Momordica charantia* L.	Ears of 8-old male ICR mice (*n* = 5/group)	Intradermal injection (solution) 0.5 mg per site	Swelling ↓ Ear weight ↓ Ear thickness ↓ Microabscess ↓	[189]
Total phenolic extract of *Momordica charantia* L. leaf	*Momordica charantia* L.	Ears of 8-old male ICR mice (*n* = 5/group)	Intradermal injection (solution) 0.5 mg per site	IL-1β^+^ ↓ Leukocyte ↓ Neutrophil ↓ Microabscess ↓	[189]
Jumihaidokuto	Pharmaceutical-grade traditional Japanese (*kampo*) medicine	Ears of 7- to 8-week-old male Sprague-Dawley rats	Oral administration (solution) 0.5 g/10 mL/kg x2	Ear thickness ↓ Inflammation ↓	[191]

↓—decrease.

**Table 2:** Application of plant phenolics in various types of acne—human studies.

Compound/Extract	Natural Sources	Study Model	Dosage	Results/Effect	References
			**Rosacea**		
EGCG	Tea, chocolate, red wine	Volunteers with rosacea (*n* = 40)	Topical application (cream) 2.5% *w*/*w*	VEGF expression ↓ HIF-1α expression ↓ Telangiectasia ↓	[195]
*Chrysanthellum indicum DC.* extract	*Chrysanthellum indicum DC.*	Patients with moderate rosacea (*n* = 125)	Topical application (cream) 1.0% *w*/*w*	Erythema ↓ Overall rosacea severity ↓	[196]
			**Acne vulgaris**		
EGCG	Tea, chocolate, red wine	Patients with Acne vulgaris (*n* = 35)	Topical application (solution) 1 and 5 *v*/*v*	Inflammatory response ↓ NF-κB ↓ AP-1 ↓ Acne lesions ↓	[158]
Quercetin (aspasomal formulation)	Tea, onions, apples, lettuce, broccoli	Patients with Acne vulgaris (*n* = 20)	Topical application (thin-layer film containing 100 µg of quercetin)	Inflammatory lesions ↓ Comedones ↓ Total lesions ↓ Antioxidant activity ↑ Activity against Propionibacterium acnes ↑	[193,198]
Green tea extract (57.12% *w*/*w* of EGCG)	*Camelia sinensis* (L.) Kuntze	Patients with Acne vulgaris (*n* = 80)	Oral supplementation (1500 mg, 3 capsules per day)	Inflammatory lesions count on the nose, forehead, cheek, periorally and on the chin ↓ Total lesions count on the forehead and cheek ↓	[200]

↑—increase; ↓—decrease.
